# Electroablation: a method for neurectomy and localized tissue injury

**DOI:** 10.1186/1471-213X-14-7

**Published:** 2014-02-16

**Authors:** José Moya-Díaz, Oscar A Peña, Mario Sánchez, Daniela A Ureta, Nicole G Reynaert, Consuelo Anguita-Salinas, Gonzalo Marín, Miguel L Allende

**Affiliations:** 1 FONDAP Center for Genome Regulation, Facultad de Ciencias, Universidad de Chile, Casilla 653, Santiago, Chile; 2 Departamento de Ciencias Biológicas, Facultad de Ciencias Biológicas, Universidad Andrés Bello, Santiago, Chile; 3 Laboratorio de Neurobiología y Biología del Conocer, Facultad de Ciencias, Universidad de Chile, Santiago, Chile; 4 Facultad de Medicina, Universidad Finis Terrae, Santiago, Chile

**Keywords:** Axotomy, Neurectomy, Tissue ablation, Regeneration, Inflammation, Zebrafish

## Abstract

**Background:**

Tissue injury has been employed to study diverse biological processes such as regeneration and inflammation. In addition to physical or surgical based methods for tissue injury, current protocols for localized tissue damage include laser and two-photon wounding, which allow a high degree of accuracy, but are expensive and difficult to apply. In contrast, electrical injury is a simple and inexpensive technique, which allows reproducible and localized cell or tissue damage in a variety of contexts.

**Results:**

We describe a novel technique that combines the advantages of zebrafish for *in vivo* visualization of cells with those of electrical injury methods in a simple and versatile protocol which allows the study of regeneration and inflammation. The source of the electrical pulse is a microelectrode that can be placed with precision adjacent to specific cells expressing fluorescent proteins. We demonstrate the use of this technique in zebrafish larvae by damaging different cell types and structures. Neurectomy can be carried out in peripheral nerves or in the spinal cord allowing the study of degeneration and regeneration of nerve fibers. We also apply this method for the ablation of single lateral line mechanosensory neuromasts, showing the utility of this approach as a tool for the study of organ regeneration. In addition, we show that electrical injury induces immune cell recruitment to damaged tissues, allowing *in vivo* studies of leukocyte dynamics during inflammation within a confined and localized injury. Finally, we show that it is possible to apply electroablation as a method of tissue injury and inflammation induction in adult fish.

**Conclusions:**

Electrical injury using a fine microelectrode can be used for axotomy of neurons, as a general tissue ablation tool and as a method to induce a powerful inflammatory response. We demonstrate its utility to studies in both larvae and in adult zebrafish but we expect that this technique can be readily applied to other organisms as well. We have called this method of electrical based tissue ablation, electroablation.

## Background

Induced and localized tissue injury is a powerful tool widely used to study biological processes, such as regeneration and inflammation. A wide range of tissue injury approaches have been employed both in regeneration and inflammation studies. Injury techniques used previously include surgical methods, such as transection and crush injury (reviewed in [1]), laser-based wounding [2-5], two photon microscopy [6], and nanoknives for microscale axon cutting [7]. Despite the high accuracy of laser and two photon-based wounding methods, these techniques have drawbacks such as requiring sophisticated equipment, and being labor-intensive and time-consuming. Other tissue injury approaches involve injection of toxins (reviewed in [8]), tissue specific expression of toxins [9], and tissue specific expression of an enzyme, which converts a nontoxic prodrug into a cytotoxic agent [10,11]. The main disadvantage of genetic approaches and other high precision techniques is the fact that in contexts like inflammation or even regeneration, such experimental models seem unrealistic considering recent evidence obtained on single axon axotomy [12].

In contrast, electrical injury provides a simple and inexpensive method for tissue injury, which also allows modulating the extent of damage produced by modifying current intensity and number of pulses. Thus, in contrast to surgery based methodologies, the extent of damage produced by electrical injury and its reproducibility across different experiments does not rely entirely on the skill of researcher, but also on the selected parameters for the electrical pulses.

Electrical damaging of tissues involves thermal, electroporation, and electrochemical interactions [13]. Once an electrical pulse is applied to a tissue, all its components are affected, however cell membranes appear to be the most vulnerable structure. Intense electric fields drive reorganization of lipids, which results in membrane permeabilization, a process called electroporation. Depending on many physical variables, electroporation can be transient or permanent [14]. While electroporation leads to the formation of transient pores in the cell membrane, Joule heating destabilizes the entire lipid bilayer [15]. Since cell membranes are composed of a lipidic bilayer held together by hydration forces, even small increments in cell membrane temperature result in complete loss of lipid bilayer integrity [16]. In addition, strong electrical fields alter the conformation of membrane proteins, as they undergo conformational changes in order to orientate their dipole moment in the direction of the field [13]. This effect, called electroconformational denaturation, is especially strong in voltage gated ion channels [17]. Since the force of an electric field drops off quadratically with distance from the focal point, electrical injury is a suitable approach circumscribing the damage to a small area of tissue [18-21].

Electrical injury has been widely used to induce damage in the adult nervous system [22,23]. However, despite the advantages offered by electrical injury to investigate the molecular and cellular basis of regeneration and inflammation *in vivo*, these studies are hampered in most cases by the opacity of the tissue. Thus, most of our current knowledge of these events comes from *in vitro* assays and analysis of fixed tissues. The optical transparency of zebrafish during embryonic and larval stages, and the availability of transgenic lines labeling different cell types, make it possible to study the behavior of almost any cell type during regeneration and inflammation *in vivo*. In this animal model, it was discovered that tissue injury induces the generation of a tissue-scale gradient of hydrogen peroxide that is required for leukocyte recruitment to wounds [24]. Furthermore, recent work in zebrafish shows that hydrogen peroxide generated upon tissue injury promotes axon growth after skin damage [12]. Furthermore, zebrafish cells and tissues regenerate robustly, a feature that allows the study of regeneration and inflammation processes in the living animal.

Our laboratory has been studying cell death, regeneration and inflammation in neuromasts of the posterior lateral line (pLL) of zebrafish larvae. The zebrafish lateral line is composed of sensory organs called neuromasts, which are distributed on the body surface. Neuromasts consist of a core of mechanosensory hair cells surrounded by support cells. Hair cells of neuromasts are innervated by neurons that form the pLL nerve which are localized in a cranial ganglion just posterior to the ear [25]. One of our goals involves the study of regeneration in a single neuromast and to examine the relationship between mechanosensory cell regeneration and innervation by the pLL nerve. Since previous approaches involved the use of heavy metals [26] or aminoglycosides [27] that cause damage by toxicity to cells, all lateral line neuromasts are damaged, hampering the study of localized damage and regeneration in a single isolated organ.

In the present work, we developed an electrical based method for neurectomy and tissue ablation, which allowed us to precisely sever nerves and to ablate small areas of tissue in a simple and reproducible way in both larvae and adult fish. The protocol can be adapted to study inflammation induced by damage, degeneration of axons and cells, and their regeneration after damage. We foresee that it will also be of utility in other model organisms where it is of interest to damage a small area of tissue in order to observe the cellular dynamics that accompany the wounding and/or regenerative process.

## Results

### A simple methodology for electrical tissue injury

Our aim was to develop a simple protocol for inducing localized tissue damage for the study of inflammation and regeneration of different cell types in zebrafish. We have taken advantage of the numerous transgenic lines available that label organs and tissues with fluorescent proteins, and that these can be readily visualized in the live zebrafish due to the optical transparency of the embryos and larvae.

**Figure 1:**
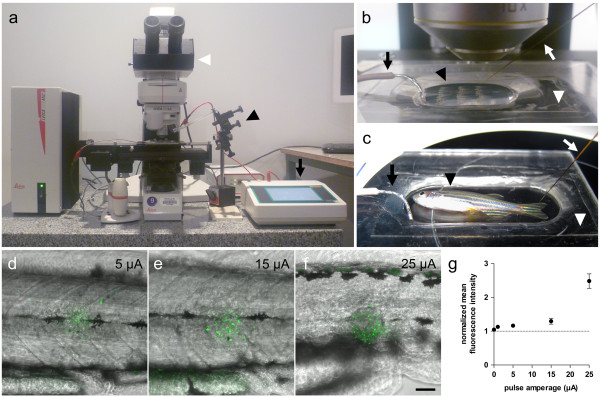
**Electroablation setup and dependence of tissue damage on amperage. (a-c)** Experimental setup for electroablation in zebrafish larvae **(b)** and adults **(c)**. **(a)** Current pulses are generated by a precision current source (black arrow), which is connected to the sample by the microelectrode and a ground wire. The microelectrode is held by a micromanipulator (black arrowhead) and zebrafish are visualized under a fluorescence microscope (white arrowhead). **(b)** Close-up view of experimental setup for electroablation in larvae. The microelectrode (white arrow) enters the agarose through one side and a ground wire is connected to the other side (black arrow). Zebrafish larvae are mounted in a drop of agarose dissolved in E3 (black arrowhead) in the central depression of the acrylic plate (white arrowhead). **(c)** Adult zebrafish (black arrowhead) are positioned in an acrylic plate (white arrowhead) with a larger depression and with a small amount of E3. The microelectrode (white arrow) touches the caudal fin and the ground wire is immersed in the E3 medium (black arrow). **(d-f)** Merge of acridine orange (green) and bright field images showing progressive expansion of acridine orange stain as amperage increases. Pulses of different amperages (5 – 25 μA) were applied for 1 second, and acridine orange stain was performed 2 hpi. **(g)** Quantification of acridine orange fluorescence shows a direct relationship with applied amperage. Average fluorescence intensity was measured within a 50 μm radius surrounding the electroablation site. Data was normalized for each larva by the average fluorescence intensity measured in an adjacent uninjured area. Values are presented as a normalized average ± SEM from 15–21 larvae per condition, from three independent experiments. Scale bar, 50 μm.

In order to induce tissue damage, we modified procedures for delivering precise current pulses developed previously for iontophoresis of neural transport tracers [28]. In our experimental setup, a precision current source (Figure 1a, black arrow) was configured to deliver electrical pulses of the desired amperage, and they were applied to zebrafish larvae with a microelecrode held by a micromanipulator (Figure 1a, black arrowhead). During the process, zebrafish are mounted (see below) and visualised under a fluorescence microscope to be able to see the cells or tissues of interest by virtue of their fluorescent protein expression (Figure 1a, white arrowhead). This allows placing the microelectrode tip at the desired location. Since the surface of the microelectrode is completely insulated with exception of the tip, it is possible to apply a pulse of current at a single point. The diameter of the microelectrode is less than 1 μm at the tip, which, together with the positioning offered by the micromanipulator, minimizes the tissue damage derived from placement of the microelectrode.

Zebrafish larvae are mounted in a drop of low melting point agarose dissolved in E3 medium (Figure 1b, black arrowhead) in an acrylic plate (Figure 1b, white arrowhead) that has a central depression. The microelectrode enters in the agarose through one side (Figure 1b, white arrow) while the ground wire is immersed in the agarose on the opposite side (Figure 1b, black arrow) to generate an electrical field through the larva. Similarly, adult zebrafish (Figure 1c, black arrowhead) are positioned in an acrylic plate with a larger depression (Figure 1c, white arrowhead), without the need of agarose, and a small amount of E3 is added to allow current conduction. The microelectrode is positioned over the target tissue (Figure 1c, white arrow) and the ground wire is immersed in the E3 medium (Figure 1c, black arrow).

To analyse the effect of pulse amperage on cell death in electroablated tissue we applied pulses of different amperages in the trunk of zebrafish larvae and performed acridine orange staining. As shown in Figure 1d-f, the application of pulses ranging from 5 μA to 25 μA produced cell damage in skin cells and progressive destruction of deeper tissues. Quantification of acridine orange staining reveals that higher amperages induced death in a larger number of cells compared with that induced by electroablation at lower amperages (Figure 1g). Thus, pulse amperage can be used to calibrate the extent of damage inflicted to tissues by electroablation.

### Electroablation as a tool for neurectomy in zebrafish

To test electroablation as a suitable methodology for neurectomy, we used the *TgBAC(neurod:EGFP)* transgenic fish that express the Enhanced Green Fluorescent Protein (EGFP) in the posterior lateral line (pLL) nerves [29]. The tip of the microelectrode was brought into contact with an anesthetized larva embedded in low melting point agarose and then a current pulse of 17 μA was applied for 1.5 seconds. Electrical injury based neurectomy of the pLL nerve had no effect on survival of larvae after seven days (data not shown). Pulses of lower amperage (~ 15 μA) were unable to completely sever the pLL axons, while higher amperages (~ 20 μA) induced extensive tissue damage. The pulse duration was also important, as 2 second-long pulses elicited aberrant trajectories in regenerated nerves (data not shown). For this reason, proper calibration of the experimental protocol is recommended in order to determine the lowest amperage and pulse times necessary to achieve complete neurectomy in all larvae while preserving the integrity of surrounding tissues.

**Figure 2:**
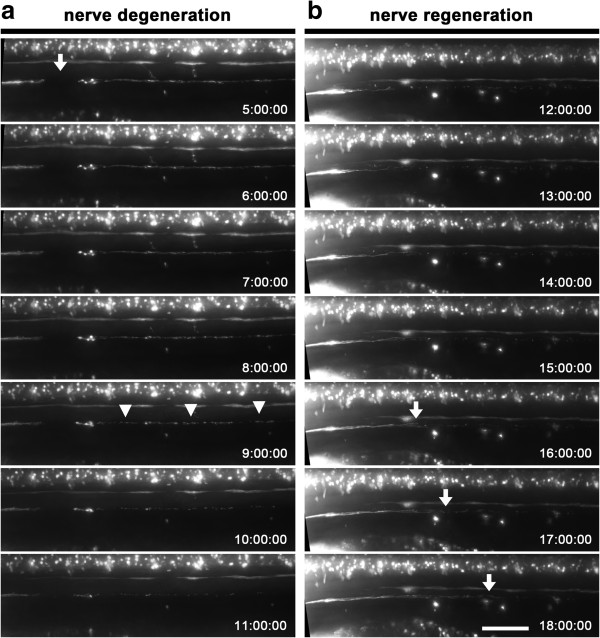
**Neurectomy, degeneration and regeneration of the posterior lateral line (pLL) nerve. (a)** Degeneration of a neurectomized pLL nerve after electrical injury. Transgenic *TgBAC(neurod:EGFP)* larvae with a labeled pLL nerve were neurectomized by applying a 17 μA pulse of current for 1.5 seconds. At 5 hpi a neurectomized larva was mounted in agarose for time-lapse imaging for 7 hours (anterior to the left). Fragmentation of the detached nerve fragment proceeds by a breakdown of the axons into many small sections simultaneously throughout the axotomized nerve (arrowheads). Note the intact contralateral pLL nerve which is visible (above the electroablated nerve, slightly out of focus) in all the images. **(b)** Regeneration of an axotomized pLL nerve. Transgenic *TgBAC(neurod:EGFP)* larvae was treated and imaged as before. In this case, the neurectomized larva was mounted for imaging for 6 hours starting at 12 hpi to examine regeneration of the pLL nerve. Note the progressive regeneration of the pLL nerve by elongation of the remaining axon stumps (arrows) as degeneration of the distal part of the axotomized pLL nerve has concluded. Scale bar, 50 μm; times in hh:mm:ss.

Neurectomy by electroablation completely severs all axons of the pLL nerve leaving a gap of 85 ± 5 μm (n = 10; three independent experiments) at the site of the lesion (Figure 2a, arrow). To examine the behavior of pLL nerve axons after neurectomy, we captured time-lapse images of the trunk of *TgBAC(neurod:EGFP)* fish five hours after pLL nerve electroablation (Figure 2a). The detached (distal) part of the pLL nerve begins to disintegrate from 8 to 11 hours post injury, hpi (Figure 2a, arrowheads), while the proximal part of the electroablated nerve retracts towards the ganglion in a process similar to acute axonal degeneration. Time-lapse imaging of axotomized larvae at later times (Figure 2b) shows growth of the pLL nerve through the site of injury and further caudally once the remaining fragments are completely cleared. Note that degeneration of the distal part of the pLL nerve is still ongoing when the regenerating nerve crosses the site of axotomy. Regeneration of the pLL nerve is completed by approximately 25 hpi. Interestingly, the dynamics of regeneration of an neurectomized pLL nerve by using electroablation are similar to those reported previously for two-photon axotomy [30].

### Electroablation applied to pLL neuromast injury

Our laboratory is interested in understanding the mechanisms involved in neuromast regeneration [26,31]. Previous studies have shown that mechanosensory hair cells in neuromasts are able to regenerate, while tools used to damage neuromast hair cells have been based on exposure to heavy metals [26] or aminoglicosides [27]. These approaches have proven to be useful for regeneration studies but they have the drawback that they involve widespread exposure of other larval tissues to the toxicants. Unlike chemical based approaches, electroablation allows the induction of a localized damage to a neuromast, making it feasible to study single neuromast regeneration.

**Figure 3:**
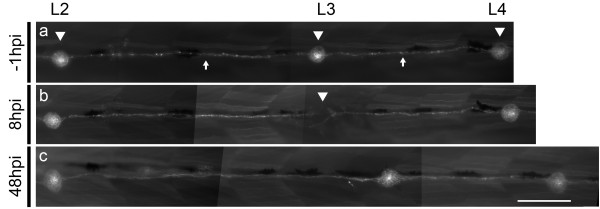
**Ablation and regeneration of a single posterior lateral line neuromast. (a-c)***Tg(cxcr4b:mRFP)* fish show labeling of posterior lateral line neuromasts and interneuromastic cells. **(a)** Lateral view of 72 hpf control larva showing intact lateral line neuromasts (arrowheads) connected by interneuromastic cells (arrows). **(b)** Two 8 μA pulses of 2 seconds each were applied to the L3 posterior lateral line neuromast. Complete dissapearance of the neuromast’s cells is observed (arrowhead) while adjacent neuromasts (L2 and L4) and interneuromastic cells remain intact. **(c)** The same larva is shown at 48 hpi, showing the regenerated L3 neuromast. Scale bar, 100 μm.

As a proof of principle for localized single neuromast ablation, we applied two 8 μA pulses for 2 seconds each in the L3 pLL neuromast of *Tg(cxcr4b:mRFP)* transgenic larvae, in which pLL neuromasts and interneuromastic cells express the red fluorescence protein [32]. As Figure 3a shows, intact neuromasts have a rosette-like structure (Figure 3a, arrowheads) and are connected to each other by interneuromastic cells (Figure 3a, arrows). Figure 3b shows the trunk of the same larva shown in Figure 3a at 8 hpi. Complete disappearance of the L3 neuromast is observed (Figure 3b, arrowhead) while adjacent neuromasts, L2 and L4, as well as most of the surrounding interneuromastic cells, remain intact. Neuromast electroablation leaves a 55.8 ± 26.3 μm (n = 20; three independent experiments) gap among interneuromastic cells.

**Figure 4:**
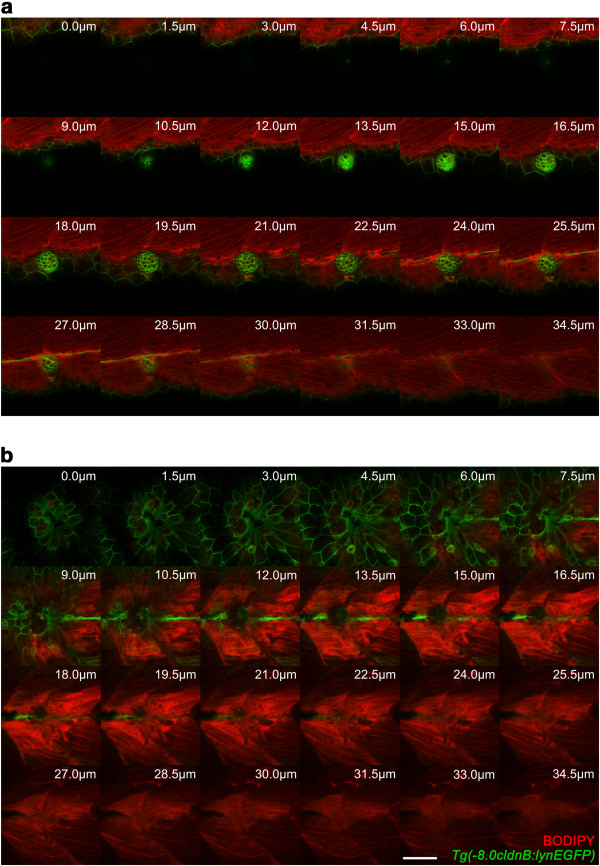
**Extent of damage inflicted to tissues by neuromast electroablation.** Transgenic *Tg(-8.0cldnb:lynEGFP)* fish, which express EGFP in lateral line cells and epithelial cells of the skin, were stained with BODIPY. Stained larvae were then mounted in agarose and subjected to electroablation of the L3 neuromast. Confocal images of electroablated and uninjured control larvae were acquired 20 minutes after injury with 1.5 μm of separation between z-axis optical slices. **(a)** Sequential z-axis confocal images of an uninjured larva are shown. The intact neuromast (green) exhibits a rosette-like structure and BODIPY-TR (red) allows visualization of underlying muscular tissue. **(b)** Images acquired as in **(a)** of a larva with an electroablated neuromast showing local loss of cells in the skin, disconnection of interneuromastic cells, destruction of neuromast cells and complete loss of the rosette-like structure. BODIPY-TR staining also shows a gap in muscular tissue to a depth of 22.5 μm from the skin surface; deeper sections appear unperturbed. Values indicate distance froom skin surface towards the inside of the larva in μm. Scale bar, 50 μm.

To further analyze the degree of damage to the surrounding tissues at the injury site, we used a contrast dye (BODIPY-TR) in *Tg(-8.0cldnb:lynGFP)* transgenic larvae [33] that express EGFP in all cell types of the lateral line and epithelial cells of the skin (Figure 4). Optical sections of the electroablated area (Figure 4b), compared to control fish (Figure 4a), show that electroablation generates tissue disruption that can be detected between the epidermis and underlying cells up to 28 μm deep, including some muscle cells. In spite of the damage inflicted by electroablation to cells beyond the lateral line cells, we observed regeneration of the ablated neuromasts (Figure 3c). While, by 24 hpi, none of the electroablated larvae exhibit regeneration of damaged neuromasts, by 48 hpi these values increase to 29.2 ± 2.2% (n = 100; three independent experiments) and by 72 hpi to 58.2 ± 4.4% (n = 100; three independent experiments). These results demonstrate that electroablation can be used to inflict localized damage to sensory organs and to study their regeneration.

### Tissue electroablation as a method to induce inflammation

Since it is well described that tissue injury induces inflammation [34,35], we aimed to apply electroablation as a suitable method to induce leukocyte recruitment to focally damaged tissues. We used the neurectomy and tissue electroablation protocols described above but now using transgenic lines in which immune cells express fluorescent proteins. Thus, we could now visualize the behavior of leukocytes during inflammation and resolution of inflammation in these contexts. Moreover, we aimed to quantify the resulting inflammation, as electroablation could be used for genetic or drug screens that use inflammation as a readout. Our results (see below) indicated that direct quantification of the number of leukocytes recruited to the site of nerve and neuromast electroablation were not feasible due to the great number of leukocytes clustering at the injury site. Thus, the degree of inflammation was instead measured as the average intensity of fluorescence of immune cells in a circular area of 50 μm radius around the injury site.

**Figure 5:**
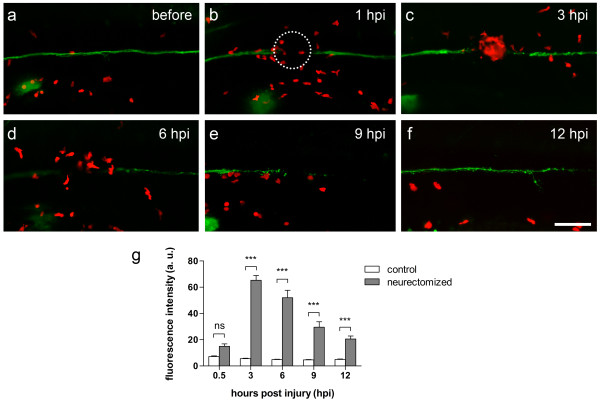
**Neutrophil recruitment induced by nerve neurectomy. (a-f)** A larva obtained from a cross of *TgBAC(neurod:EGFP)* and *Tg(lyz:DsRED2)* transgenic fish labels the posterior lateral line nerve in green and leukocytes in red. **(a)** Control larva showing an intact lateral line nerve and few leukocytes near the nerve. One hour after lateral line nerve neurectomy **(b)**, leukocytes migrate and accumulate at the lesion site **(b and c)**. From 6 hpi, the number of recruited leukocytes decreases and resolution of inflammation takes place. **(d-f)**. The number of recruited leukocytes continues to decrease as the lateral line nerve regenerates. **(g)** Quantification of average fluorescence intensity within a 50 μm radius surrounding the neurectomy site (panel **b**, dotted circle) shows leukocyte recruitment and subsequent inflammation resolution. Data are presented as average ± SEM from 17 larvae per condition from two independent experiments. Comparisons were performed by using a repeated measurement two-way ANOVA, with Bonferroni’s post test. ***, *p* < 0.001; ns, *p* > 0.05. Scale bar **(a-f)** 100 μm. hpi, hours post-injury; a. u., arbitrary units.

To examine the inflammatory response induced by neurectomy of the pLL nerve, we used a *Tg(BACneurod:EGFP; lyz:DsRED2)* compound transgenic line, labeling the pLL nerve in green [29] and myeloid leukocytes (mainly neutrophils) in red fluorescence [36], respectively. In these fish, we observed that pLL nerve neurectomy induces recruitment of leukocytes to the site of electroablation, forming a cell cluster at the electroablation site within the first 3 hpi (Figure 5a-c). At 6 hpi, the number of leukocytes recruited to the site of neurectomy decreases as inflammation resolution proceeds (Figure 5d). The number of localized leukocytes further decreases as degeneration of the neurectomized nerve proceeds (Figure 5e), and is still decreasing by 12 hpi, when the regenerating nerve has reached the third neuromast of the pLL (L3) (Figure 5f). To quantify the degree of inflammation elicited by neurectomy, we acquired images and measured mean red fluorescence intensity in a circular area centered at the electroablation site (Figure 5b, dotted circle). As is shown in Figure 5g, myeloid leukocytes accumulate at the site of neurectomy during the first 3 hours and then their number decreases for the next 9 hours as inflammation resolution takes place. Concomitant with the infiltration of neutrophils into the site of neurectomy, macrophages are also recruited to the site of injury during the first 2 hours after damage (Additional file 1). Compound transgenic fish, *Tg(mpeg1:EGFP; neurod:TagRFP)*, which harbor macrophages that express GFP [37] and a pLL nerve labeled with red fluorescent protein, were subjected to pLL neurectomy by application of a 17 μA pulse for 1.5 seconds. A time series of macrophage infiltration dynamics into the area surrounding the electroablation site shows accumulation of a large number of macrophages as a result of the induced damage.

**Figure 6:**
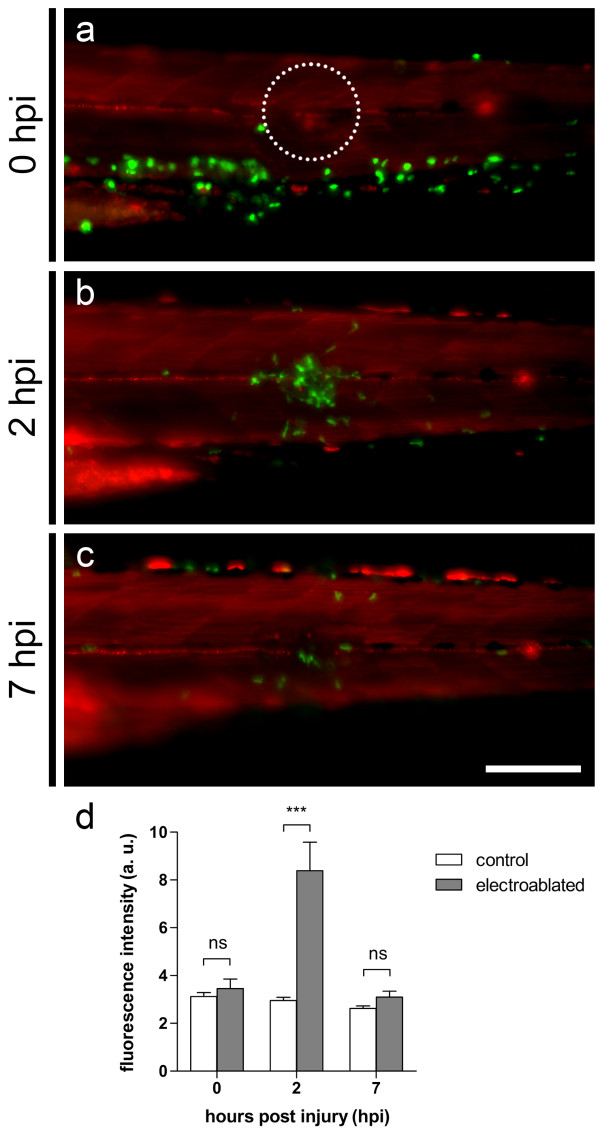
**Neuromast electroablation induces an inflammatory response.** Compound transgenic *Tg(cxcr4b:mRFP; mpx:GFP)* fish harboring neutrophils expressing green fluorescent protein and neuromasts labeled in red were used to study neutrophil inflammation induced by neuromast electroablation. Two 2 second 8 μA pulses were applied to induce damage to the pLL neuromast. **(a-c)** The trunk of a larva subjected to neuromast electroablation is shown with anterior to the left. **(a)** Immediately after electroablation, most neutrophils are present in the caudal hematopoietic tissue (CHT). **(b)** At 2 hpi, a large number of neutrophils have specifically migrated to the site of damage. **(c)** By 7 hpi, the number of neutrophils at the site of electroablation has diminished, suggesting resolution of inflammation is taking place. **(d)** Quantification of mean fluorescence in a 50 μm radius around the site of electroablation (panel a, dotted circle) as a measure of neutrophil recruitment. Data are presented as average ± SEM from 12 larvae per condition and two independent experiments. Comparisons were performed by using a repeated measurement two-way ANOVA, with Bonferroni’s post test. ***, *p* < 0.001; ns, *p* > 0.05. Scale bar **(a-b)** 100 μm. hpi, hours post-injury; a. u., arbitrary units.

Similarly, it is also possible to examine the inflammatory response induced by the electroablation of a pLL neuromast. We used *Tg(cxcr4b:mRFP; mpx:GFP)* compound transgenic fish labeling pLL neuromasts in red [32] and neutrophils in green fluorescence [38] to study neutrophil inflammation. In these fish, we observed that pLL neuromast electroablation induces potent recruitment of neutrophils to the site of electroablation (Figure 6). Recruited neutrophils form a cell cluster at the site of electroablation by 2 hours after the injury (Figure 6b). Seven hours after neuromast electroablation, the number of leukocytes recruited to the site of damage has decreased, suggesting that resolution of neutrophil-mediated inflammation has taken place (Figure 6c). *In vivo* time lapse imaging of an electroablated larva (Additional file 2), shows how neutrophils migrate interstitially, mostly from the caudal hematopoietic tissue (CHT), to the damaged neuromast. There are also neutrophils migrating from the dorsal ridge to the site of electroablation. Interestingly, those neutrophils in the CHT that are close to the damaged neuromast, exhibit directional migration to the wound, while those neutrophils which are farther away from the ablation site show low or no motility at all. Accordingly, quantification of neutrophil-mediated inflammation (Figure 6d) shows a rapid increment in the number of recruited neutrophils during the first 2 hours after neuromast electroablation, and a decrease to values similar to those of control larvae over the next 5 hours.

### Electroablation applied to other tissues

**Figure 7:**
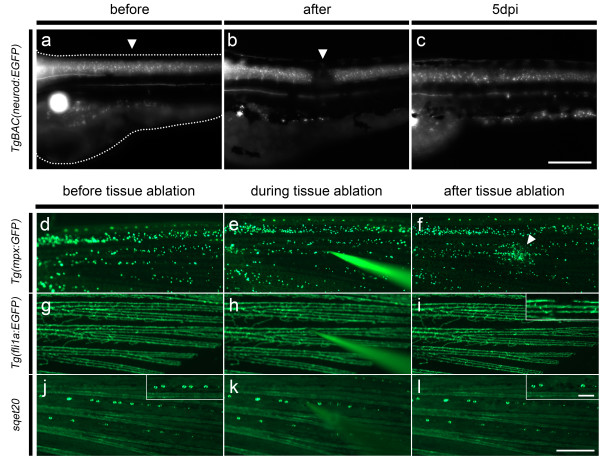
**Electroablation applied to spinal cord injury in larvae and tissue damage in the caudal fin of adult fish. (a-c)** 72 hpf *TgBAC (neurod:EGFP)* transgenic larvae were subjected to spinal cord injury. **(a)** The intact spinal cord before electroablation is shown. The dotted lines show the outline of the larva. **(b)** A 1 second 25 μA pulse was applied in the spinal cord (arrowhead), leaving a gap and rendering larvae unable to move their tails. **(c)** The regenerated spinal cord at 5 dpi. **(d-i)** Damaging of different tissues in caudal fin of adult fish by application of a 2 second 25 μA pulse of current. **(d-f)** Induction of neutrophil recruitment in the caudal fin of adult *TgBAC(mpx:GFP)* transgenic fish. A large number of neutrophils recruited to the site of damage is observed by 2 hpi **(f)**. **(g-i)***Tg(flia:EGFP)* transgenic adult fish in which blood vessels are labeled in green were subjected to electroablation in a single blood vessel **(h)**, leaving a gap in it, as shown in **(i)** (see inset for higher magnification). **(j-l)** Caudal fin neuromast electroablation in *sqet20* transgenic fish, which possess neuromasts labeled in green. **(j)** Intact fin neuromasts are shown. Within the line of five neuromasts shown in the inset, note positioning of the microelectrode over the second pair of neuromasts **(k)**. **(l)** After electroablation, the pair of neuromasts has been destroyed and a gap in the GFP pattern can be observed at that position (see inset for details). Note that the electrode is easily observable during electroablation facilitating its positioning **(e, h and k)**. Scale bars: a-c, 100 μm; d-l, 500 μm; and i, j, and l insets 100 μm. dpi, days post-injury.

We further aimed at applying our electrically based tissue injury method to other tissues and also in adult fish. Our results with pLL nerve neurectomy led us first to test electroablation in the central nervous system. We developed a spinal cord injury protocol which allowed us to completely sever the spinal cord in zebrafish larvae. At 72 hpf, *TgBAC(neurod:EGFP)* transgenic fish, which express EGFP in spinal cord neurons and axons, were anesthetized and mounted in agarose dissolved in E3, and a 25 μA pulse of 1 second was applied in their spinal cord. An intact larva before electroablation is shown in Figure 7a. Figure 7b shows the same larva after spinal cord electroablation. This protocol eliminates nervous tissue leaving a gap (Figure 7b, arrowhead) of 84.92 ± 0.037 μm (n = 40; four independent experiments) and rendering larvae paralyzed (not shown). The same larva at 5 dpi is shown in Figure 7c; it exhibits robust regeneration as new fibers have crossed the ablation site and the larvae show recovery of mobility (not shown).

Next, we used electroablation to induce superficial tissue injury in adult fish. The application of electroablation in adults involved the use of acrylic plates larger than those used with larvae (see Methods). Adult transgenic fish were anesthetized and positioned in acrylic plates, and a 25 μA pulse of 2 seconds was applied on their caudal fin. As done before in larvae, we wished to determine if localized tissue damage inflicted by electroablation in caudal fins of adult fish elicited neutrophil recruitment to the site of injury. Adult *Tg(mpx:GFP)* transgenic fish, in which neutrophils are labeled in green, were subjected to electroablation in the caudal fin (Figure 7d-f). Before tissue injury, neutrophils are scattered in the caudal fin (Figure 7d). Local tissue injury was applied (Figure 7e) and 2 hours later, a cluster of neutrophils has formed at the lesion site (Figure 7f, arrowhead). In order to quantify neutrophil recruitment, we acquired images of electroablated caudal fins in the same transgenic fish immediately after electroablation and two hours later. Quantification of mean fluorescence intensity in a circular area with a 100 μm radius surrounding the damage point shows a significant increase (p-value = 0.0361, paired *t* test) in electroablated *vs* control fish (mean fluorescence, after injury: 3.80 ± 0.51, 2hpi: 15.48 ± 4.60; n = 9, three independent experiments).

Likewise, we induced tissue damage using two transgenic lines that label other tissues in the caudal fin of adult fish. Figure 7g shows the caudal fin of a *Tg(fli1a:EGFP)* transgenic fish, in which blood vessels are labeled in green [39]. The electrode was placed precisely on a vessel to sever it (Figure 7h). The result shows that electroablation allows specific damage to a single blood vessel (see Figure 7i, inset), leaving a gap of 127.4 ± 13.19 μm (n = 12; three independent experiments) while adjacent vessels remain intact. Similarly, electroablation allowed neuromast ablation in the caudal fin of adult fish (Figure 7j-l). The caudal fins of adult *sqet20* transgenic fish harbor green-labeled neuromasts [40], which are distributed regularly in several lines along the fin (Figure 7j). In this case, electroablation allowed specific damaging of two adjacent neuromasts (see Figure 7j inset). The microelectrode was positioned on the neuromasts (Figure 7k) and a 25 μA pulse of 2 seconds was applied, destroying both neuromasts (Figure 7l inset) with no observable alterations in adjacent neuromasts. These experiments illustrate how transgenic lines and electroablation can be easily combined to induce local damage in a tissue of interest, allowing the study of regeneration and inflammation in adult fish.

## Discussion

We describe an electrical injury method, electroablation, which will contribute to regeneration and inflammation research by providing a simple and versatile method for neurectomy, tissue ablation and inflammation induction. The transparency of zebrafish larvae and availability of fluorescent tags in specific tissues in transgenic fish, combined with the advantages of an electrical injury method (localized and precise tissue damage and modulation of the extent of the damage), will now facilitate *in vivo* analysis of regeneration and inflammation. Moreover, electroablation can also be applied to adult fish, making it possible to carry out regeneration and inflammation studies in this context.

While other methodologies allow single cell ablation with high precision or even to sever a single axon [6], our approach allows localized tissue damaged without cell type specificity. We think this can be an advantage rather than a limitation, since nerve and tissue injury generally involve non-specific damage to several cell types. Therefore, we propose electroablation as a relevant model for inducing tissue injury in a way that reflects all of the non-specific tissue damage related events. This aspect is critical for regeneration studies, since it has been reported that tissue regeneration depends on hydrogen peroxide production [12,41], which is induced by tissue damage but does not occur in response to single cell ablation [12].

Our laboratory is particularly interested in the regeneration of both the pLL nerve and neuromasts. We believe that electroablation will be of value for the study of the molecular mechanisms and the roles of different cell types involved in regeneration in both models. In spite of the high resistence of nerves and their surrounding tissues, we show successful pLL nerve neurectomy by electroablation, as a cheap and simple alternative to more sophisticated approaches, such as two-photon axotomy [6]. Similarly to previous studies performed by two-photon axotomy on the pLL nerve [12] we observed Wallerian degeneration of the detached axons after electroablation and, later, pLL nerve regeneration. In addition to pLL nerve regeneration studies, this methodology will allow the study of the mechanisms involved in hair cell re-innervation, as well as the role of inervation on hair cell regeneration in this organ system.

Using electroablation we show, for the first time, the complete ablation and regeneration of a single neuromast. Since electroablation allows single neuromast ablation, we foresee that this method will make it possible to study the mechanisms involved in single neuromast regeneration as well as the role of different cell types, such as interneuromastic cells, Schwann cells, neutrophils and macrophages in this process.

Furthermore, we show that electroablation can be adapted to be used in adult fish, allowing the study of regeneration and inflammation in adult animals. This advantage could be used to investigate the effects of chronic conditions, such as exposure to toxic compounds, on regeneration and inflammation, for example.

### Other potential applications of electroablation

In addition to peripheral neurectomy, neuromast ablation, spinal cord injury, inflammation induction and tissue damage in adult fin tissue, we foresee additional applications using this methodology. First, electroablation can be modified for use in regeneration studies as a general strategy for ablation of different tissues. Tissues and organs where electroablation should be feasible include the liver, olfactory rosettes, blood vessels, muscles, eyes, brain, and ganglia. Furthermore, electroablation could be used as a reproducible method for injury to almost any superficial tissue with the aid of the appropriate transgenic fish labeling the tissue of interest. Our method should be of use even in deep tissues with the aid of simple surgery protocols, making it possible to inflict damage in internal organs in adult fish.

Second, since electroablation allows localized tissue damage and modulation of the degree of damage, this approach can be used for inflammation studies. Different methods have been used to induce inflammation in zebrafish, which include techniques involving physical methods, such as manual tail transection [42] and laser induced wounds [5], or a genetically induced chronic inflammatory condition [43]. Our approach takes advantage of small microelectrodes and electrical pulses to induce a localized tissue injury that induces immune cell recruitment. Thus, the main advantages of this method for inflammation studies are its robustness and versatility. Since our methodology relies on electrical pulses, it allows different degrees of tissue damage, which can be achieved by means of controlling current intensity, and the number and duration of the pulses applied. This advantage could be used to investigate possible relationships between the extent of tissue damage and the inflammatory response, and furthermore, to study the mechanisms of resolution of inflammation involved in each case.

Furthermore, this approach allows repeated localized tissue damage, a useful approach for the study of the dynamics of immune cells. Since this method can be easily combined with *in vivo* time lapse imaging of immune cells (Additional file 2) and with cell tracking tools, leukocyte navigation could be studied in an inflammatory context *in vivo*, knowing exactly where the inflammatory signals will be produced*.* Thus, questions regarding signal integration, receptor desensitization and immune cell fate after successive inflammatory stimuli, aspects that remain unknown, could be addressed with the aid of this methodology.

Finally, electroablation is a powerful tool for neuroinflammation studies, as it allows nervous system injury or neurectomy as well as inflammation induction. The aforementioned advantages of this approach could be used to investigate the molecular mechanisms of immune system involvement in axon or neural regeneration.

## Conclusions

In conclusion, electroablation is a versatile tool that will facilitate the study of regeneration and inflammation in a variety of tissues in larvae and adult fish, as well as providing a simple model for neurectomy and new tools to investigate regeneration, dynamic aspects of leukocyte recruitment to wounds, and immune system involvement in regeneration.

## Methods

### Zebrafish husbandry and experimental conditions

Zebrafish (*Danio rerio*) larvae were obtained in our facility according to standard procedures [44]. We used the transgenic strains *TgBAC(neurod:EGFP)nl1*[28], *Tg(neurod:TagRFP)w69*[45], *Tg(cxcr4b:mRFP)ump1*[32], *Tg(-8.0cldnb:lynGFP)zf106*[33], *Tg(lyz:DsRED2)nz50*[36], *Tg(mpx:GFP)i114*[38], *Tg(mpeg1:EGFP)*[37], *Tg(fli1a:EGFP)y1*[39] and *Et(krt4:EGFP)sqet20* herein named *sqet20*[40]. All embryos were collected by natural spawning and raised at 28.5°C in E3 medium (5 mM NaCl, 0.17 mM KCl, 0.33 mM CaCl_2_, 0.3 mM MgSO_4_, and 0.1% methylene blue, equilibrated to pH 7.0) in Petri dishes. Pigment formation was avoided by supplementing E3 medium with PTU (Sigma) 3% from 24 hpf. Embryos and larvae were staged according to Kimmel *et al*. [46], and larval ages are expressed in hours post-fertilization (hpf). All animals used in this work were anesthetized with MS-222 (tricaine, A5040, Sigma, St. Louis, MO, USA) before each experiment. All procedures complied with guidelines of the Animal Ethics Committee of the University of Chile.

### Electroablation system components

The setup for electroablation is composed by a precision current stimulator (AM System, Model 2100; or Catalog #51595, Stoelting) that provides current pulses to a tungsten microelectrode (Catalog #UEWMGGSEBN1M, FHC Inc., Bowdoin, ME, USA) which is positioned with a micromanipulator (Narishige M-152) (see Figure 1a). Larvae are visualized with a fluorescence microscope (AS LMD, Leica) equipped with 10x lens objective. For visualization at higher magnifications, objectives of longer focal distance are needed, in order to allow the entry and movement of the microelectrode between the objective and the sample. The stimulator allowed adjustment of current, and number and duration of current pulses. Tungsten microelectrodes were insulated with epoxylite, and its final 120 μm of length were tapered at an angle ranging from 10 to 15°, so that microelectrodes were less than 1 μm of diameter at the tip. Since microelectrodes are extremely narrow, they needed some preparation to be used with our micromanipulator. We sanded down the epoxylite from the thicker part of the microelectrode and then placed it in a needle (0.80 × 40 mm, Catalog #305167, BD). This allowed us to firmly adjust the microelectrode in the pipette holder of our micromanipulator.

### Tissue injury protocols

*TgBAC(neurod:EGFP)nl1* and *Tg(neurod:TagRFP)* transgenic larvae were used for pLL nerve neurectomy and spinal cord injury, and *Tg(cxcr4b:mRFP)ump1* and *Tg(-8.0cldnb:lynGFP)* fish were used for neuromast electroablation. Larvae were anesthetized using MS-222, embedded in 0,75 or 1% low melting point agarose (BM-0130, Winkler, Spain) dissolved in E3 on acrylic plates (10.7 cm long × 7 cm wide) with a central depression and positioned using forceps before the agarose set (see Figure 1b). Both control and experimental larvae were mounted in order to avoid any manipulation-derived bias. Note that agarose concentration is important to facilitate electroablation, since lower concentrations of agarose are not strong enough to keep embedded larvae immobile, and thus hamper the penetration of tissues by the microelectrode (as occurs, for instance, in spinal cord electroablation) while, conversely, higher concentrations of agarose make it difficult to get the microelectrode into the agarose.

For pLL nerve neurectomy, the microelectrode was positioned in the region between the pLL nerve ganglion and the first neuromast. Neurectomy was carried out by applying a current pulse of 17 μA for 1.5 seconds. Immediately after a successful electroablation, a gap can be seen at the site of ablation in the pLL nerve together with GFP^+^ labeled debris, possibly corresponding to axon fragments. All larvae showing incomplete axotomy are discarded in order to avoid misleading conclusions from spared axons.

Single neuromast ablations were performed in the third neuromast of the pLL (L3) by bringing the microelectrode into contact with the neuromast and then applying two 8 μA pulses, each for 2 seconds. After current application, careful examination of each larva was performed in order to discard those larvae with partial neuromast ablation. With the exception of the time-lapse experiments, electroablated larvae were left unmounted and kept in E3. Temperature is extremely important in order to achieve optimal percentages of regenerated neuromasts with this protocol. Therefore, appropriate incubation at 28°C of larvae before and after electroablation is advised for best results.

For spinal cord injury, larvae were mounted sideways in 1% agarose to keep them immobilized while the microelectrode penetrates the tissue. The microelectrode was positioned laterally in the region depicted in Figure 7a (arrowhead), between the horizontal myoseptum and the dorsal ridge, and then pushed into the larval tissue until it reached the spinal cord. Spinal cord injury was achieved by applying a 25 μA pulse for 1 second. After electroablation all those larvae with incomplete spinal cord injury are discarded, which can be evaluated both by checking for mobility impairment (caudal to the site of damage) and by looking for surviving axons under a fluorescence microscope.

Electroablation in caudal fins of adult fish involved the use of an acrylic plate with a larger depression (see Figure 1c). Adult fish were anesthetized, and positioned on the acrylic plate with a minimal amount of E3 to allow efficient conduction (see Figure 1c). The microelectrode was positioned as can be seen in Figure 7e, h and k, and a 25 μA pulse for 2 seconds was applied. Since the entire process can be done in less than a minute electroablation does not represent a threat to the survival of adult fish.

In order to determine optimal conditions to achieve regeneration or to induce inflammation in other applications of electroablation, it is best to attempt tissue ablation and neurectomy at a low power with just one pulse, and then incrementally increase the power and number of pulses until the appropriate settings are found.

### Quantification of neutrophil-mediated inflammation and acridine orange staining

Compound *Tg(BACneurod:EGFP; lyz:DsRED2)*, for pLL nerve neurectomy, *Tg(cxcr4b:mRFP; mpx:GFP)*, for pLL neuromast ablation, and *Tg(mpx:GFP)*, for adult fin electroablation, transgenic larvae were subjected to electroablation as described in the previous section. In order to quantify leukocyte infiltration, images were acquired of the damaged site with a fluorescent stereoscope (Olympus, model MVX10) and then average fluorescence intensity was measured in a circular area (50 μm radius for quantifications in larvae, and 100 μm in adult fish) centered at the site of electroablation by using ImageJ software, version 4.2 [47]. The results are expressed in arbitrary units. Similarly, for acridine orange quantification, images were acquired from the electroablated fish using a fluorescence stereoscope (Olympus MVX10) and average fluorescence intensity was measured within a 50 μm radius around the site of electroablation and in an adjacent control area using ImageJ. Average fluorescence intensity in the damaged area was normalized by the measurement taken at the control site for each larva.

### Image processing

For imaging, larvae were anesthetized and mounted in low melting point agarose dissolved in E3. Photographs were taken with a Zeiss LSM 510 Meta confocal microscope, or an Olympus IX81 fluorescence microscope. For time-lapse imaging, we used a Zeiss Axiovert 200 M microscope equipped with a 20X lens objective and an Axiocam camera (Figure 2) or an Olympus MVX10 fluorescent stereoscope and recorded with a QImaging digital camera (Additional file 1 and Additional file 2). All images were processed with Zeiss Axiovision (Carl Zeiss Microimaging GmbH, Jena, Germany) and ImageJ software Version 4.2 [45]. As described in Figure 2a, at 5 hpi axotomized transgenic larvae (*TgBAC(neurod:EGFP)*) were anesthetized and embedded in 1% low melting point agarose dissolved in E3. Images were captured every 60 minutes for 7 hours. As described in Figure 2b, 12 hpi axotomized transgenic (*TgBAC(neurod:EGFP)*) larvae were anesthetized and embedded in 1% low melting point agarose dissolved in E3 for imaging for a total of 6 hours. Images were acquired every 60 minutes for 6 hours. As described in Additional file 1, compound transgenic fish (*Tg(mpeg1:EGFP; neurod:TagRFP)*) were neurectomized and immediately mounted in 0.75% low melting point agarose dissolved in E3. Images were captured every 15 seconds during 2 hours. As described in Additional file 2, larvae were subjected to neuromast electroablation and immediately embedded in 1% low melting point agarose dissolved in E3. Images were captured every 30 seconds for a total of 32 minutes.

### Statistical analysis

Data are presented as mean values ± SEM. Statistical analysis was performed using GraphPad Prism version 5.00 for Windows software (GraphPad Software, San Diego, CA, USA). The probability level for statistical significance was *p* < 0.05.

## Abbreviations

pLL: Posterior lateral line; CHT: Caudal hematopoietic tissue; hpf: Hours post-fertilization; dpf: Days post-fertilization; hpi: Hours post-injury; dpi: Days post-injury; GFP: Green fluorescent protein; EGFP: Rnhanced green fluorescent protein; RFP: Red fluorescent protein.

## Competing interests

The authors have declared that no competing interests exist.

## Authors’ contributions

JM developed the methodology, carried out nerve regeneration studies, participated in inflammation and neuromast regeneration studies, and helped to draft the manuscript. MS developed the neuromast ablation protocol, carried out neuromast regeneration and inflammation studies and helped to draft the manuscript. OP carried out inflammation studies, performed the statistical analysis and drafted the manuscript. DU and NR helped to carry out inflammation studies. CA carried out spinal cord injury studies. GM participated in the design of the study and development of methodology. MA conceived of the study, participated in its design and helped to draft the manuscript. All authors read and approved the final manuscript.

## Supplementary Material

Additional file 1 **Recruitment of macrophages to the site of neurectomy.** Compound transgenic fish, *Tg(mpeg1:EGFP; neurod:TagRFP)*, labeling macrophages in green and the pLL nerve in red, were subjected to pLL neurectomy by application of a 17 μA pulse for 1.5 seconds. A temporal series of images (only green channel shown) shows macrophage infiltration into the site of axotomy starting 20 minutes after electroablation. Scale bar, 200 μm. Times expressed in hh:mm:ss. Click here for file

Additional file 2 **Inflammatory neutrophils recruited towards an electroablated neuromast.** Compound *Tg(cxcr4b:mRFP; mpx:GFP)* transgenic fish which have red-labeled pLL neuromasts and green-labeled neutrophils were subjected to neuromast electroablation and immediately mounted for imaging for 32 minutes under a fluorescent stereoscope. Images were captured every 30 seconds in the green channel. Interstitial migration of neutrophils from the caudal hematopoietic tissue (CHT) to the damaged neuromasts and also from the dorsal ridge can be observed. Scale bar, 100 μm. Times are expressed as hh:mm:ss. Click here for file
