# Supplementary figures and images for: Electroablation: a method for neurectomy and localized tissue injury
Source: BMC Dev Biol. 2014 Feb 16;14:7. doi: 10.1186/1471-213X-14-7 (PMC3933190; doi:10.1186/1471-213X-14-7)

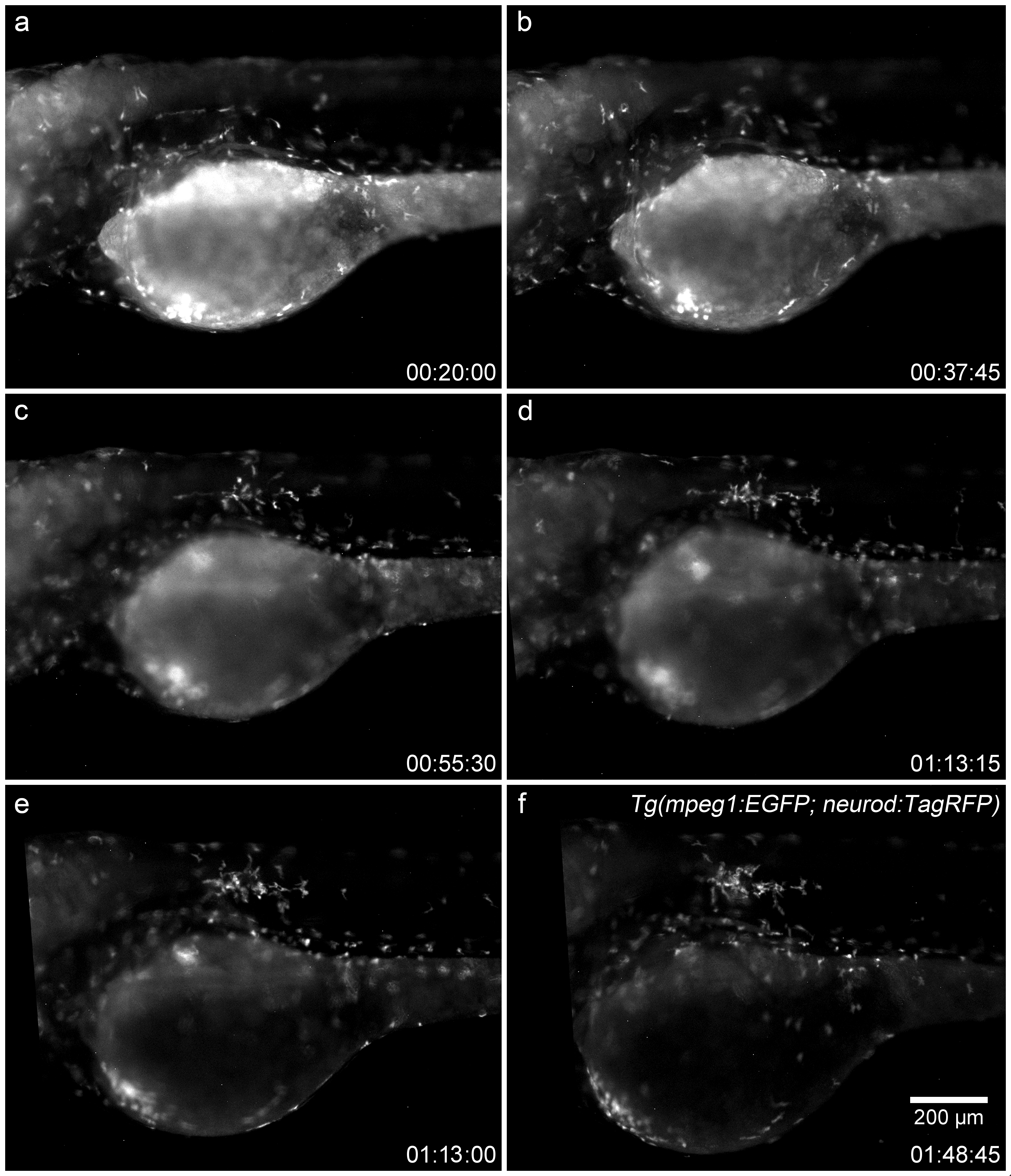

Supplement: Additional file 1 — Recruitment of macrophages to the site of neurectomy. Compound transgenic fish, Tg(mpeg1:EGFP; neurod:TagRFP), labeling macrophages in green and the pLL nerve in red, were subjected to pLL neurectomy by application of a 17 μA pulse for 1.5 seconds. A temporal series of images (only green channel shown) shows macrophage infiltration into the site of axotomy starting 20 minutes after electroablation. Scale bar, 200 μm. Times expressed in hh:mm:ss. [file 1471-213X-14-7-S1.tiff]
